# 1’,4’-*trans*-diol-ABA enhances the growth and physiological characteristics of rice (*Oryza sativa* L.) seedlings under salt stress

**DOI:** 10.1371/journal.pone.0284734

**Published:** 2025-06-02

**Authors:** Yin Jie, Shengjie Feng, Dianfeng Zheng, Naijie Feng, Hang Zhou, Xixin Huang, Anqi Huang, Rongjun Zhang, Fengyan Meng

**Affiliations:** 1 College of Coastal Agricultural Sciences, Guangdong Ocean University, Zhanjiang, Guangdong, China; 2 South China Center of National Saline-Tolerant Rice Technology Innovation Center, Zhanjiang, Guangdong, China; 3 Shenzhen Research Institute of Guangdong Ocean University, Shenzhen, Guangdong, China; University of Agriculture Faisalabad, PAKISTAN

## Abstract

Rice (*Oryza sativa* L.) is a glycophyte that can be easily poisoned by salt toxicity. 1’,4’-*trans*-diol-ABA is a crucial precursor in the biosynthesis of abscisic acid (ABA) in fungi that may alleviate salt stress in plants; however, the role of this compound on salt tolerance in rice has not yet been reported. In the present study, 1’,4’-*trans*-diol-ABA was applied to rice seedling varieties “Huanghuazhan” (HHZ) and “Xiangliangyou 900” (XLY900) to investigate the resulting tolerance against saline stress. Results demonstrated that salinity stress reduced the growth parameters of seedlings of two rice cultivars, made lower photosynthesis and fluorescence parameters. The salt treatment also increased the concentration and Na^ +^ content of malondialdehyde (MDA) of two rice cultivars, and decreased the catalase (CAT) activity, ascorbyl peroxidase (APX) and peroxidase (POD) activity, ascorbic acid (AsA) and glutathione (GSH) of rice seedlings of two varieties. The results showed that the exogenous addition of 1’,4’-*trans*-diol-ABA decreased salt-induced reactive oxygen species (ROS) accumulation and oxidative stress and improved the morphological characteristics of both rice cultivars, compared to cultivars without the addition of 1’,4’-*trans*-diol-ABA. Furthermore, changes in different phytohormones in rice seedlings were triggered by 1’,4’-*trans*-diol-ABA treatment. Simultaneously, the Na^ + ^/K^ +^ ratios in the shoots and roots of treated plants were lower than in the non-treated plants, which may be due to the higher K^ +^ concentration observed in treated plants under saline water irrigation conditions. In conclusion, this study revealed that 1’,4’-*trans*-diol-ABA may perform a signiﬁcant function in increasing crop salt-stress tolerance.

## Introduction

The 2020–22 triennial was marked by the unprecedented challenges posed by the COVID-19 pandemic as well as the socio-economic impacts arising from multiple risks and threats such as climate change, environmental degradation, trade frictions, and regional conflicts. Despite hopes that the world would emerge from the COVID-19 pandemic and food security would begin to improve, world hunger has and is predicted to further increase as a sequela of the epidemic. The latest estimates by the United Nations Food and Agriculture Organization (FAO) put the global hunger figure for 2021 between 702 and 828 million people [1]. This estimate implies that, since the onset of the pandemic, the increase in the number of undernourished people in the world has practically eroded all progress made during the preceding decade, bringing the world back to hunger levels that prevailed in 2005. Therefore, it is imperative to increase crop yields, especially rice (*Oryza sativa* L.), the staple food of more than half the world’s population [2].

Rice is the most salt-sensitive cereal, which means it cannot survive in saline-alkali soil for long [3]. Increasing soil salinity has a negative effect on plant growth, survival, and productivity. Previous studies have found that osmotic stress signaling initiates after salt stress. Osmotic stress inhibits the expansion of root and shoot cells within minutes due to the reduced turgor pressure under salt stress [4,5]. The stress signal is transduced rapidly, at the speed of the sound, through different signaling cascades from the root to the shoot tissue to close the stomata and minimize water loss, which halts shoot metabolism [6]. The mechano-sensitive ion channels of the leaf guard cells sense the drop in xylem pressure and participate in stomatal closure, reducing CO_2_ exchange and photosynthetic assimilation [7].

Second, for osmotic adjustment, plants start to intake salt ions from the soil, which then enter different root and shoot cells [8]. The signaling cascade and response to the ionic imbalance initiate later due to the slow accumulation of sodium ions (Na^+^) in shoot tissues beyond a threshold, with the corresponding inhibition of photosynthesis [9]. This affects the rate of electron transport through photosystems and results in increased reactive oxygen species (ROS), mainly $O_{2}^{▪-}$ , production [10]. Mitochondrial respiration is another primary source of salt-induced ROS production. Over-reduction of the ubiquinone pool during salt stress allows electrons to leak from mitochondrial electron transport chain complexes I and III to molecular oxygen, resulting in $O_{2}^{▪-}$ production [11,12].

The chemical formula of 1’,4’-*trans*-diol-ABA is C_15_H_22_O_4_. In 1973, 1’,4’-*trans*-diol-ABA was first chemically synthesized by Walton et al. [13]. It was then identified as an essential precursor for ABA synthesis in many fungi [14]. Multiple reports have inferred that 1’,4’-*trans*-diol-ABA may have similar functions to ABA on account of their similar structures [15]. Abscisic acid (ABA) is a widely studied phytohormone and its role in ameliorating abiotic stress in plants is well established [16], so it is aptly called a stress hormone [17]. The exogenous application of ABA can promote moderate stomatal closure, inhibit excessive water reduction, increase chlorophyll content, protect Photosystem II (PSII) to alleviate stress-induced photoinhibition, increase antioxidants, and alleviate membrane lipid peroxidation [18–22]. Although 1’,4’-*trans*-diol-ABA is also found in a few higher plants, this does not mean it plays the identical role as ABA.

Research into the anti-salt effects of 1’,4’-*trans*-diol-ABA is still in its infancy and little study about its physiological effects on crops has been undertaken. In this study, the appropriate concentration of 1’,4’-*trans*-diol-ABA was determined first based on the morphology of rice seedlings. Subsequently, anti-salt metabolites in rice shoots were detected. The potential impact of 1’,4’-*trans*-diol-ABA as a plant growth regulator in rice tolerance and growth recovery in response to salinity and ethylene stress was determined based on the agronomic, physiological, morphological, hormone levels, and ion concentrations of two different rice cultivars.

## Materials and methods

### Materials

1’,4’-*trans*-diol-ABA was obtained from the Chengdu Institute of Biology, Chinese Academy of Sciences. It was dissolved in 95% ethanol to prepare a 4 g·L^–1^ stock solution. The working concentration (0.5, 1, 2, 4, 8 mg·L^–1^) was obtained by dilution with water. All the reagents were of analytical grade.

### Screening the optimal concentration of 1’,4’-*trans*-diol-ABA

Two indica rice cultivars, “Huanghuazhan” (HHZ, salt-sensitive) and “Xiangliangyou 900” (XLY900, also known as “Chaoyouqianhao”, salt tolerant [23]), were selected for soil pot culture. This was conducted in a greenhouse in 2021 at the Guangdong Ocean University, Guangdong Province, China. First, rice seeds (HHZ, XLY900) were sterilized by soaking with 3% H_2_O_2_ for 20 min, washed thoroughly in ddH_2_O, and germinated for 2 d at 30 °C in the darkness. Uniform seedlings were selected and sown in pots (19 ×  14 ×  17 cm) containing a 3 kg mixture of sand and laterite (1:3 v/v). At the 3-leaf stage, the two varieties were divided into seven groups each variety: control, 50 mmol·L^–1^ NaCl, 1’,4’-*trans*-diol-ABA (0.5, 1, 2, 4, and 8 mg·L^–1^). Next, different concentrations of 1’,4’-*trans*-diol-ABA were sprayed on the leaf surface of each group and pure ddH_2_O was used to inoculate the control and NaCl plants. There were five replicates in each group. Based on our preliminary experiments and previous studies [24], the salt solution concentration (50 Mm) was selected. After foliar spraying 1’,4’-*trans*-diol-ABA for 48 h, to simulate the salt stress conditions, 50 mM NaCl solution was poured into each pot except for the control treatment; that is, 9 g NaCl per barrel of potted plants and the concentration of total salt solution is about 0.3%. Seedling growth was photographed on day 4 after the addition of salt.

### Growth characteristics

The foliage of rice seedlings was sprayed with 2 mg·L^–1^ 1’,4’-*trans*-diol-ABA and collection was on days 0, 1, 4, 7, and 10. Eight groups were established in total: control, 50 mmol·L^–1^ NaCl, 2 mg·L^–1^ 1’,4’-*trans*-diol-ABA, 2 mg·L^–1^ 1’,4’-*trans*-diol-ABA +  50 mmol·L^–1^ NaCl for each variety. The plant height, stem diameter, leaf area, root length, and total dry weight were measured with a ruler, vernier caliper, leaf area meter, and an electronic analytical balance. The dry weight was obtained after oven drying at 105 °C for 30 min and then at 85 °C for 72 h, when a constant weight was obtained. The roots were scanned by a desktop scanner (Epson CORP, Japan), and then the images were analyzed by WinRZIZO root analysis software (Regent Instruments, Inc, Quebec, Canada) to obtain the root length, root surface area, root volume and root average diameter.

### Photosynthetic activity

The net photosynthetic rate (*P*_*n*_), transpiration rate (*T*_*r*_), intercellular CO_2_ concentration (*C*_*i*_), and stomatal conductance (*gs*) of the ﬂag leaves were recorded following the method of [25] using a portable photosynthesis system (LI-6400, LI-COR, USA) on a sunny day between 9:00 a.m. to 12:00 p.m at day 10. The CO_2_ concentration in the leaf chamber was 400 μmol·mol^ − 1^, the air velocity was 500 μmol·s^ − 1^, the light intensity was 1000 μmol·m^ − 2^s^ − 1^, the leaf temperature was 32 ±  1 °C, and the relative air humidity was between 70% and 80%.

### Chlorophyll content

The chlorophyll (*Chl a*, *Chl b*, carotenoids, and total chlorophyll) was extracted from fresh ﬂag leaves (0.1 g) by mixing with 10 mL of 95% alcohol and incubating at 24 h in the dark at room temperature (25 °C) at days 10. The absorbance of the sample was then measured at 665, 649, and 470 nm via UV-VIS spectrophotometer (GENESYS 180, Thermo Fisher, USA) to estimate the chlorophyll content according to the methods of [26], with slight modiﬁcations. The following equations were used for the calculation of pigment amounts:


$$\text{Chlorophyll a }(C_{a})=13.95A_{665}-6.88A_{649}$$

$$\text{Chlorophyll b }(C_{b})=24.96A_{649}-\;7.32A_{665}$$

$$\text{Carotenoids }=\frac{1000A_{470}-2.05C_{a}-104C_{b}}{245}$$

$$\text{Total Chlorophyll content }=C_{a}+C_{b}+\text{Carotenoids}$$

### Malondialdehyde (MDA) and soluble protein content

According to the method of a previous study [27], 0.5 g of leaf sample was ground with 10 mL of 10% trichloroacetic acid (TCA), centrifuged at 10,000 ×  g for 10 min and 1 mL of supernatant was mixed with 2 mL of 0.6% thio barbituric acid (TBA) in another tube. This mixture was heated in boiling water (100 °C) for 15 min. After rapid cooling, the mixture was centrifuged again at 10,000 ×  g for 10 min and the absorbance of the supernatant was determined at 450, 532, and 600 nm. The MDA concentration was calculated according to the following equation:


$$\text{C μmol = 6.452 × }\left({\text{A}}_{\text{532}}{\text{- A}}_{\text{600}}\right)\text{- 0}{\text{.559 × A}}_{\text{450}}$$

The level of soluble protein was measured using the Coomassie brilliant blue method [28]. The absorbance of the supernatant was measured at 595 nm after 2 min of reaction. Then, the protein content in the sample was determined according to the standard curve with bovine serum albumin (BSA).

### Hormone content

The phytohormones from rice seedlings leaves were analyzed according to a previously described method [29]. Briefly, 0.1 g of fresh leaf samples was collected at 24 hours and immediately stored in liquid nitrogen. Then, the samples were ground into powder in liquid nitrogen in the laboratory. An aliquot of each sample was precisely weighed and transferred to an Eppendorf tube. After the addition of 1000 μL of extract solution (50% acetonitrile in water, precooled at – 40 °C, containing isotopically-labelled internal standard mixture), the samples were vortexed for 30 s and sonicated for 5 min in an ice-water bath, and homogenized at 40 Hz for 4 min. The vortexing and sonication were repeated three times. Then, the samples were centrifuged at 12000 rpm for 15 min at 4 °C. A 900 μL aliquot of the supernatant was evaporated to dryness under a gentle stream of nitrogen and was reconstituted in 90 μL of 10% ACN/H_2_O. All the samples were vortexed for 30 s and sonicated for 5 min in an ice-water bath. After the samples were centrifuged at 12000 rpm for 15 min at 4 °C, the clear supernatant was subjected to UHPLC-MS/MS (SCIEX, USA) analysis.

### Antioxidant system

Leaf samples (0.5 g) were homogenized in 10 mL of 50 mM phosphate buffer (pH 7.8) containing 1% polyvinylpolypyrrolidone (PVP). These samples were then centrifuged at 10,000 ×  g for 10 min at 4 °C for superoxide dismutase (SOD), catalase (CAT), ascorbate peroxidase (APX) and peroxidase (POD) assay. CAT, APX, SOD and POD activity was measured according to previously reported methods [30]. Soluble protein was determined by the method of [31] using BSA as the standard. The determination of GSH and AsA content followed the method described by [32].

### Ion content

At 10 days of salt stress, the Na^ + ^, K^ + ^, and Ca^2 +^ content was determined in 150 mg dry mass (DW) of leaves and roots following the method of [24]. Samples were digested in 6 mL of HNO_3_/HClO_4_ (4:1, v/v) and the supernatants were analyzed after clarifying the digestate. Na^ +^ and Ca^2 +^ content was measured by ICP-AES (Prodigy XP, Leeman, USA) and the K^ +^ content was measured by a flame photometer (Sherwood M410, UK).

### Statistical analysis

Statistical analyses were performed using the SPSS Statistics 25. Origin 2021 was used for picture drawing. Comparisons between two groups were performed by one-way analysis of variance (ANOVA). Comparisons between multiple groups were performed by ANOVA followed by Duncan’s multiple range test.

## Results

### Effects of 1’,4’-*trans*-diol-ABA on agronomic traits of rice under salinity stress

**Fig 1:**
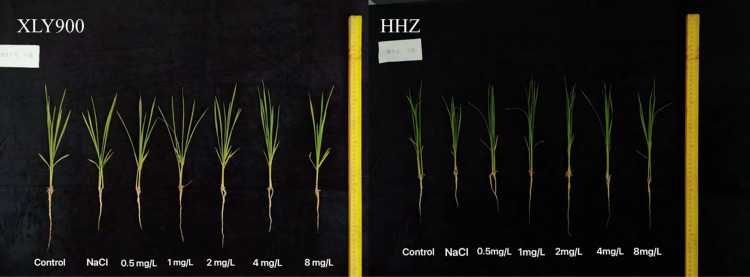
Morphology of rice seedlings treated with different concentrations of 1’,4’-*trans*-diol-ABA from 0.5 to 8 mg·L–1 under salt stress at day 4. XLY900 = Xiangliangyou 900, HHZ =  Huanghuazhan.

The growth of the rice seedlings with exposure to 1’,4’-*trans*-diol-ABA at 0.5, 1, 2, 4, and 8 mg·L^–1^ (Fig 1) was studied to determine the optimal concentration. The plant height of the two varieties of rice markedly decreased under salt stress. However, compared with the NaCl group, different concentrations of 1’,4’-*trans*-diol-ABA could reverse the decrease in plant height induced by salt, and 2 mg·L^–1^ had the greatest ameliorating effect. Accordingly, for the optimum effect and to minimize reagent use, 2 mg·L^–1^ was chosen for further experiments.

Salinity stress reduced the plant height, stem diameter, leaf area, and plant dry weight for both rice cultivars. For HHZ, as the salinity stress increased from the control (0 mM NaCl) to the 50 mM NaCl solution, the plant height significantly decreased by 7.30%–22.07% between days 0, 1, 4, 7, and 10. Additionally, the stem diameter significantly decreased by 7.06%–14.29%, the leaf area decreased by 0.47%–51.97%, the shoot DW decreased by 2.46%–26.65%, and the root DW decreased by 8.35%–30.67% compared to the control group. For XLY900, the plant height decreased by 8.87%–24.20%, the stem diameter by 12.33%–15.73%, the leaf area by 4.29%–57.02%, the shoot DW by 12.64%–26.72%, and the root DW by 24.48%–40.19% compared to the control group.

**Fig 2:**
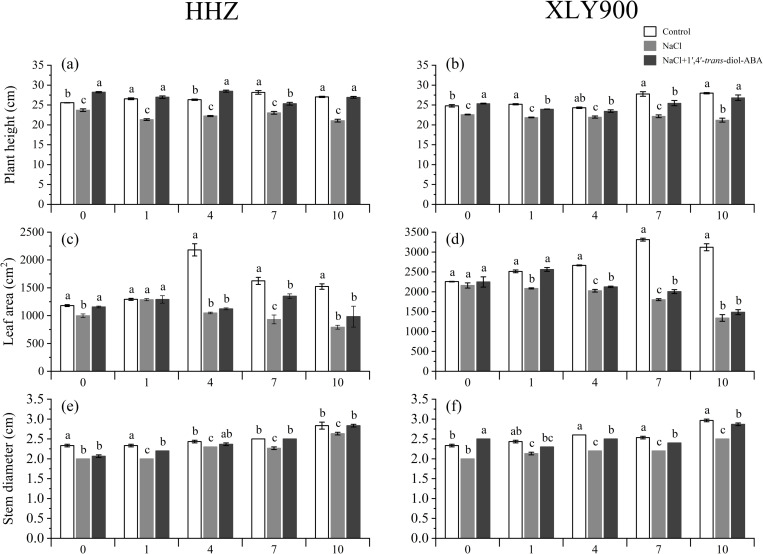
Effect of exogenous 1’,4’-*trans*-diol-ABA on the growth parameters of HHZ and XLY900 at days 0, 1, 4, 7, and 10 under saline conditions. Plant height of (a) HHZ and (b) XLY900; stem diameter of (c) HHZ and (d) XLY900; leaf area of (e) HHZ and (f) XLY900. Different lowercase letters indicate significant differences according to Duncan’s test (*P <  0.05). Values are mean ±  SE (*n* =  3).

**Fig 3:**
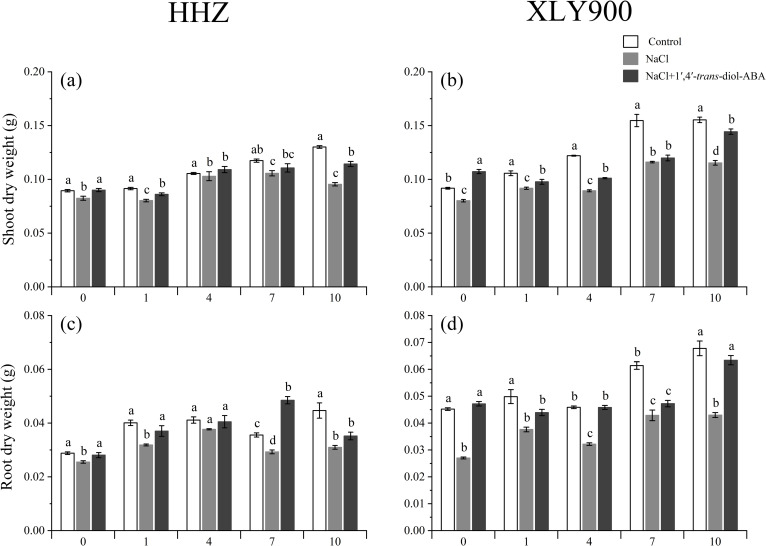
Effect of exogenous 1’,4’-*trans*-diol-ABA on the dry weight of HHZ and XLY900 at days 0, 1, 4, 7, and 10 under saline conditions. Shoot DW of (a) HHZ and (b) XLY900; root DW of (c) HHZ and (d) XLY900. Different lowercase letters indicate significant differences according to Duncan’s test (*P <  0.05). Values are mean ±  SE (*n* =  3).

The application of 1’,4’-*trans*-diol-ABA improved the morphological parameters of the two rice cultivars. For HHZ, the plant height was enhanced by 9.99%–28.04%, the stem diameter by 3.33%–10.29%, the leaf area by 0.38%–44.95%, the shoot DW by 4.76%–19.77%, and the root DW by 7.43%–65.43% compared to the plants without 1’,4’-*trans*-diol-ABA application. Similarly, for XLY900, the application of 1’,4’-trans-diol-ABA increased the plant height by 6.83%–26.57%, the stem diameter by 9.09%–25%, the shoot DW by 3.2%–33.80%, and the root DW by 10.19%–74.48% compared to the plants without 1’,4’-*trans*-diol-ABA application (Fig 1). Thus, the plant height, stem diameter, leaf area, and plant DW confirmed the positive effect of 1’,4’-*trans*-diol-ABA on rice seedling growth under salt stress (Figs 2 and 3).

### Effects of 1’,4’-*trans*-diol-ABA on the root morphology of rice under salt stress

**Fig 4:**
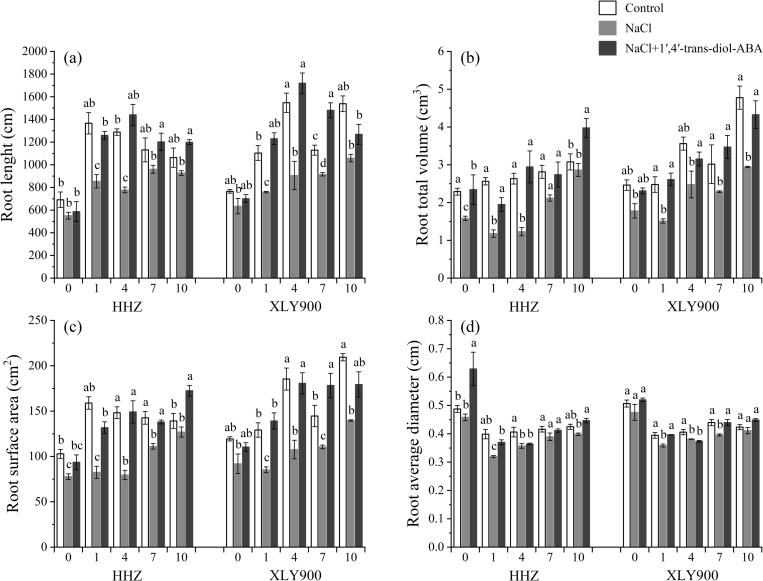
Effect of exogenous 1’,4’-*trans*-diol-ABA on the root morphologies of HHZ and XLY900 at days 0, 1, 4, 7, and 10 under saline conditions. (a) Root length in HHZ and XLY900, (b)root total volume, and in HHZ and XLY900, (c) root surface area in HHZ and XLY900, (d) root average diameter in HHZ and XLY900. Different lowercase letters indicate significant differences according to Duncan’s test (*P <  0.05). Values are mean ±  SE (*n* =  3).

Treatment with NaCl significantly inhibited root growth. The root length significantly decreased by 15.05%–39.60% for HHZ (Fig 4a) and 17.09%–41.47% for XLY900 (Fig 4b) from 0–10 days compared to the control. Simultaneous treatment with NaCl and 1’,4’-*trans*-diol-ABA led to a significant increase in root length by 6.48%–85.09% for HHZ and 10.47%– 89.90% for XLY900 as compared to NaCl treatment alone. 1’,4’-*trans*-diol-ABA also ameliorated the decrease in total volume, surface area, and average root diameter under saline. In 1’,4’-*trans*-diol-ABA–treated roots, the total volume, surface area, and average diameter increased remarkably by 29.24%–139.29% (Fig 4c), 20.33%–87.25% (Fig 4e), and 1.88%–37.27% (Fig 4g) for HHZ, respectively, and by 27.30%–72.56% (Fig 4d), 20.01%–67.85% (Fig 4f), and 9.17%–11.02% (Fig 4h) for XLY900, respectively, compared to NaCl treatment alone. As expected, pretreatment with 1’,4’-*trans*-diol-ABA significantly alleviated NaCl- inhibited root growth.

### Effects of 1’,4’-*trans*-diol-ABA on photosynthesis-related attributes of rice under salinity stress

**Fig 5:**
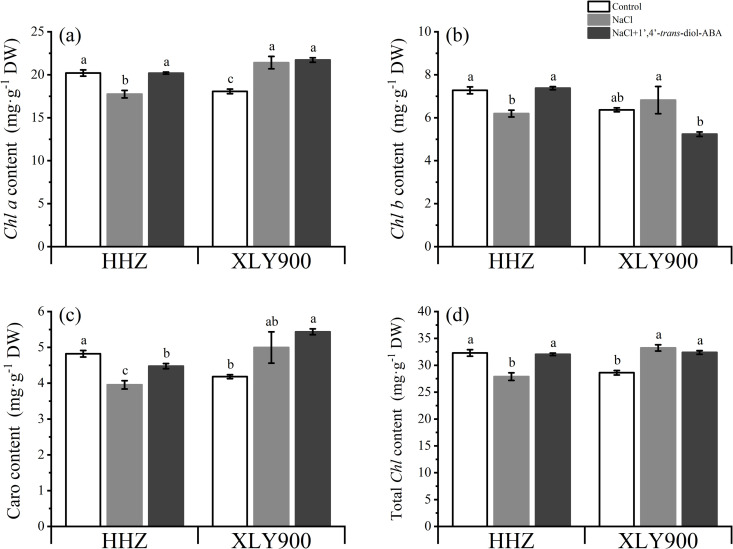
Effect of exogenous 1’,4’-*trans*-diol-ABA on photosynthetic pigments contents of HHZ and XLY900 at days 10 under saline conditions. (a) *Chl a* in HHZ and XLY900, (b) *Chl b* in HHZ and XLY900, (c) caro in HHZ and XLY900, (d) total chlorophyll content in HHZ and XLY900. Different lowercase letters indicate significant differences according to Duncan’s test (*P <  0.05). Values are mean ±  SE (*n* =  3).

Compared with the control, salinity significantly decreased the *Chl a*, *Chl b*, and the total chlorophyll content of HHZ at day 10, and increased the *Chl a, Chl b*, carotenoid, and total chlorophyll content of XLY900 at days 10. However, foliar spraying with 1’,4’-*trans*-diol-ABA alleviated the salt stress. As shown below (Fig 5), 1’,4’-*trans*-diol-ABA significantly increased HHZ *Chl a* by 13.77%, *Chl b* by 19.05%, carotenoids by 13.26%, and total chlorophyll by 14.86% at day 10. But for XLY900 increased by 1.45% (*Chl a*), 8.76% (carotenoid), and decreased by 23.25% (*Chl b*), 2.52% (total chlorophyll content) after applying 1’,4’-*trans*-diol-ABA, respectively.

**Fig 6:**
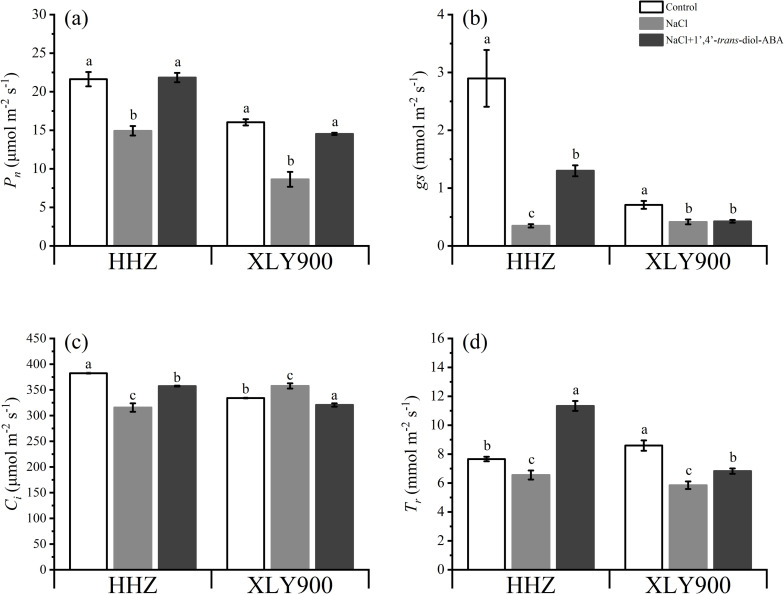
Effect of exogenous 1’,4’-*trans*-diol-ABA on photosynthetic gas exchange parameters of HHZ and XLY900 at days 10 under saline conditions. (a) *P*_*n*_ in HHZ and XLY900, (b) *gs* in HHZ and XLY900, (c) *C*_*i*_ in HHZ and XLY900, (d) *T*_*r*_ in HHZ and XLY900. Different lowercase letters indicate significant differences according to Duncan’s test (*P <  0.05). Values are mean ±  SE (*n* =  3).

Similarly, the exposure of plants to saline-alkali conditions inhibited photosynthesis in rice leaves as indicated by the significant reduction in *P*_*n*_, *gs*, *C*_*i*_, and *T*_*r*_ by 30.97%, 88.01%, 17.44%, and 14.40% in HHZ, respectively, and on *P*_*n*_, *gs*, and *T*_*r*_ by 46.13%, 41.55%, and 31.96% in XLY900, respectively. In contrast, 1’,4’-*trans*-diol-ABA application significantly increased *P*_*n*_, *gs*, *C*_*i*_, and *T*_*r*_ by 46.21%, 270%, 13.20%, and 72.85% in HHZ, and increased *P*_*n*_, *gs*, and *T*_*r*_ by 68.27%, 2.33%, and 16.70% in XLY900, respectively (Fig 6).

### Effects of 1’,4’-*trans*-diol-ABA on the antioxidant system, MDA, and soluble protein content of rice under salinity stress

**Fig 7:**
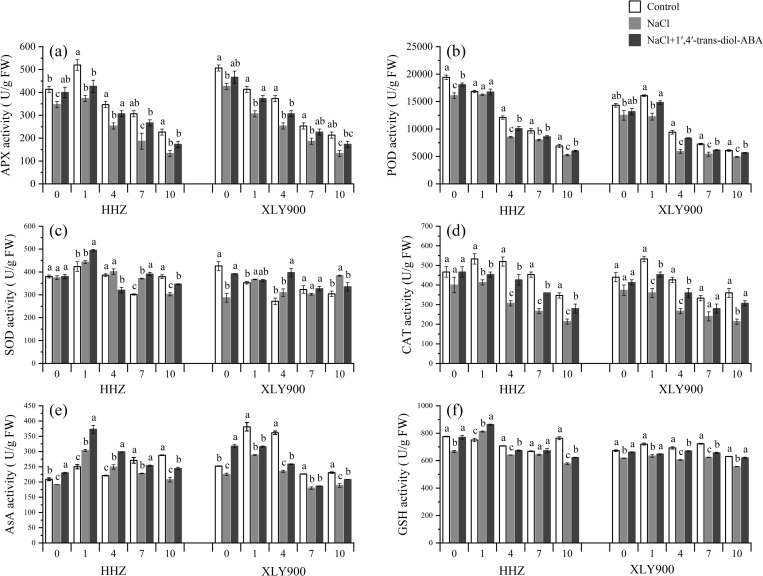
Effect of exogenous 1’,4’-*trans*-diol-ABA on antioxidant system of HHZ and XLY900 at days 0, 1, 4, 7, and 10 under saline conditions. (a) APX in HHZ and XLY900, (b) POD in HHZ and XLY900, (c) SOD in HHZ and XLY900, (d) CAT in HHZ and XLY900, (e) AsA in HHZ and XLY900, (f) GSH in HHZ and XLY900. Different lowercase letters indicate significant differences according to Duncan’s test (*P <  0.05). Values are mean ±  SE (*n* =  3).

To prevent damage, the antioxidant defenses of plants must keep active oxygen under control (Fig 7). An analysis of enzymatic activity revealed that, after exposure to NaCl, there was a signiﬁcant decrease in the total activity of CAT, APX, and POD. Compared with the control, the activity of CAT, APX, and POD in both rice cultivars following 1’,4’-*trans*-diol-ABA leaf spraying was significantly increased, and there was a significant increase in AsA and GSH content. For HHZ, 1’,4’-*trans*-diol-ABA treatment increased the antioxidant enzyme activity by 9.68%–39.13% (CAT), 14.38%–42.86% (APX), 3.08%–18.03% (POD), 17.76%–223.01% (AsA), and 4.61%–15.43%(GSH), compared to the control. There was a similar trend in XLY900; its antioxidant enzyme activity increased by 10.71%–43.75% (CAT), 9.38%–30% (APX), 9.72%–40.84% (POD), 3.56%–41.21%(AsA), and 2.08%–11.48(GSH).

**Fig 8:**
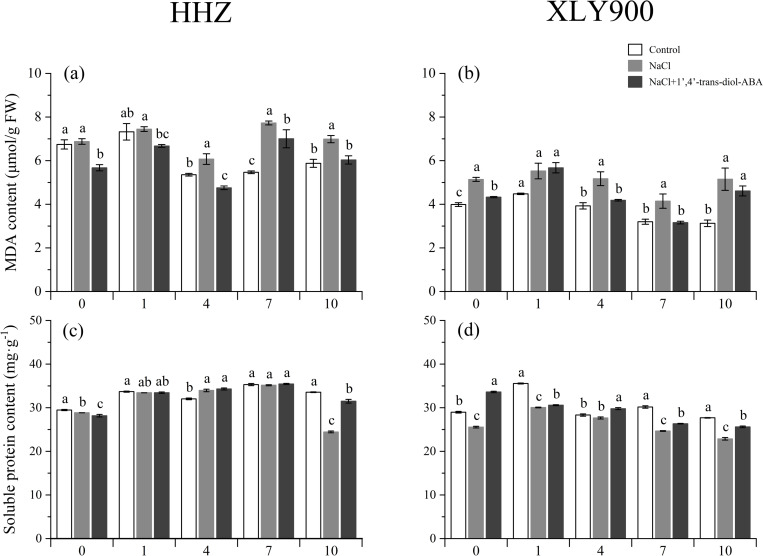
Effect of exogenous 1’,4’-*trans*-diol-ABA on MDA and soluble protein content of HHZ and XLY900 at days 0, 1, 4, 7, and 10 under saline conditions. MDA content in HHZ (a) and XLY900 (b), soluble protein content in HHZ (c) and XLY900 (d). Different lowercase letters indicate significant differences according to Duncan’s test (*P <  0.05). Values are mean ±  SE (*n* =  3).

The terminal products of lipid peroxidation are “aggressive” substances, such as aldehydic secondary products (malondialdehyde, 4-hydroxy-nonenal, 4-hydroxy-hexenal, and acrolein); these are markers of oxidative stress [33]. Compared with the control group, MDA content increased by 1.70%–41.36% (HHZ) and 23.40%–64.72% (XLY900) under NaCl treatment at 0,1,4,7,10 day, indicating that NaCl exacerbated the peroxidation of membrane lipids. 1’,4’-*trans*-diol-ABA decreased the MDA content by 9.39%–21.68% (HHZ) and 10.47%–73% (XLY900) relative to the NaCl group, which indicated that 1’,4’-*trans*-diol-ABA alleviated oxidation damage to some extent (Figs 8a and b). Additionally, soluble protein is the substance that maintains osmotic pressure [34], and its concentration increased with the addition of 1’,4’-*trans*-diol-ABA. Compared with the NaCl group, soluble proteins increased by 0.03%–28.74% (HHZ) and 1.77%–31.52% (XLY900; Figs 8c and d).

In this study, rice that received 1’,4’-*trans*-diol-ABA showed higher SOD and POD activity. This was beneﬁcial for scavenging ROS and alleviating membrane lipid peroxidation, as demonstrated by the lower MDA content. Therefore, the results indicated that 1’,4’-*trans*-diol-ABA could enhance the antioxidant ability of the rice cultivars to decrease salt damage.

### Effects of 1’,4’-*trans*-diol-ABA on the ion content of rice under salinity stress

**Fig 9:**
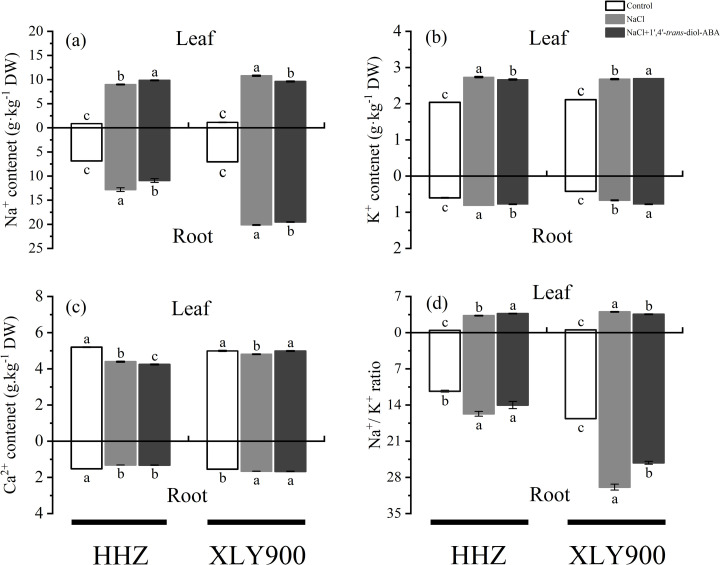
Effect of exogenous 1’,4’-*trans*-diol-ABA on ion contents of HHZ and XLY900 at days 10 under saline conditions. (a) Na^ +^ content in HHZ and XLY900, (b) K^ +^ content in HHZ and XLY900, (c) Ca^2 +^ content in HHZ and XLY900, (d) Na^ + ^/K^ +^ ratio in HHZ and XLY900. Different lowercase letters indicate significant differences according to Duncan’s test (*P <  0.05). Values are mean ±  SE (*n* =  3).

The Na^ +^ concentration in roots and leaves was significantly enhanced 9-fold (leaf) and 87.06% (root) in HHZ and 8.6-fold (leaf) and 1.8-fold (root) in XLY900 under NaCl treatment. The Na^ +^ concentration was significantly decreased by 11.01% in leaf, 2.98% in root of XLY900, and 61% in root of HHZ, which treatment with 1’,4’-*trans*-diol-ABA in the leaves (Fig 9a). Compared with NaCl treatment, K^ +^ showed the same trend but then significantly decreased by 2.44% (leaf) and 4.88% (root) in HHZ and increased by 0.75% (leaf) and 15.84% (root) in XLY900 after spraying with 1’,4’-*trans*-diol-ABA (Fig 9b). On the other hand, Ca^2 +^ decreased by 15.44% (leaf), 13.46% (root) in HHZ, and 3.69% in the leaf of XLY900 after NaCl stress. Also, after spraying 1’,4’-*trans*-diol-ABA, it increased by 0.35% in the roots of HHZ and 3.68% (leaf) and 3.68% (leaf), 0.52% (root) in XLY900 (Fig 9c). After application of 1’,4’-*trans*-diol-ABA, the Na^ + ^/K^ +^ ratio decreased significantly by 10.79% (root) in HHZ and 11.71% (leaf), 15.75% (root) in XLY900 (Fig 9d). This demonstrated that 1’,4’-*trans*-diol-ABA plays a crucial role in ion homeostasis in plants.

### Effects of 1’,4’-*trans*-diol-ABA on phytohormones of rice under salinity stress

For HHZ, NaCl treatment increased the ACC (3.16%), ABA (70.5%), IAA (38.97%), SA (6.47%), and tZ (6.49%) levels compared to the control group. Treatment with 1’4’-*trans*-diol-ABA either further enhanced these levels by 23.71% (ABA), 53.48% (N6), 132.44% (tZr), 23.31% (tZ), 75.32% (cZ) or decreased them by 6.52% (ACC), 30.08% (IAA), and 6.00% (SA). N6-isopentenyladenosine (N6), *trans*-Zeatin-riboside (tZr), *trans*-Zeatin (tZ), and *cis*-Zeatin (cZ) are different types of cytokinin that are widespread in higher plants [35,36]. Interestingly, for XLY900, salinity treatment resulted in the same increasing trend for ACC (31.24%), ABA (98.05%), IAA (25.9%), and tZ (44.86%) but a decrease in SA (5.38%) content compared to the control group. Regardless, treatment with 1’,4’-*trans*-diol-ABA enhanced these levels by 17.19% (ABA) and 9.23% (IAA). However, 1’,4’-*trans*-diol-ABA did not increase the N6, tZr, tZ, and cZ levels, instead decreasing these by varying degrees. These interesting results revealed that under salinity-induced stress, both rice cultivars tried to achieve homeostasis in terms of phytohormone production (Tables 1 and 2).

**Table 1:** Effect of exogenous 1’,4’-*trans*-diol-ABA on the ACC, ABA, IAA, and SA content of HHZ and XLY900 at 24h under saline conditions.

Cultivars	Treatment	ACC (nmol/kg FW)	ABA (nmol/kg FW)	IAA (nmol/kg FW)	SA (mol/kg FW)
HHZ	Control	321.44 ± 5.18bc	36.93 ± 2.50d	49.88 ± 0.32b	163.08 ± 8.194.23a
	NaCl	331.60 ± 7.29bc	62.97 ± 1.98b	69.31 ± 2.96a	173.63 ± 6.404.02a
	tdABA+ NaCl	309.98 ± 6.65c	69.31 ± 2.96a	48.47 ± 3.13b	163.28 ± 3.626.62a
XLY900	Control	302.81 ± 21.54b	37.99 ± 0.98c	36.86 ± 1.27b	124.32 ± 2.813.50a
	NaCl	397.39 ± 14.24a	75.25 ± 3.93b	46.33 ± 2.04a	117.64 ± 5.263.66a
	tdABA+ NaCl	323.44 ± 40.91ab	88.18 ± 5.7a	50.61 ± 1.81a	117.44 ± 2.268.66a

Values are the mean ±  SE (*n* =  3). Different lowercase letters indicate significant differences according to Duncan’s test.

**Table 2:** Effect of exogenous 1’,4’-*trans*-diol-ABA on the N6, tZr, tZ, and cZ content of HHZ and XLY900 at 24h under saline conditions.

Cultivars	Treatment	N6 (nmol/kg FW)	tZr (nmol/kg FW)	tZ (nmol/kg FW)	cZ (nmol/kg FW)
HHZ	Control	1.11 ± 0.08b	1.50 ± 0.08b	0.16 ± 0.01c	0.12 ± 0.02ab
	NaCl	0.98 ± 0.10bc	1.18 ± 0.25bc	0.17 ± 0.01bc	0.08 ± 0.01b
	tdABA+ NaCl	1.50 ± 0.06a	2.75 ± 0.22a	0.20 ± 0.01a	0.13 ± 0.02a
XLY900	Control	5.46 ± 0.37a	9.11 ± 1.00a	0.14 ± 0.01b	0.25 ± 0.03a
	NaCl	2.42 ± 0.25b	3.80 ± 0.17b	0.21 ± 0.01a	0.14 ± 0.01b
	tdABA+ NaCl	1.73 ± 0.31b	2.49 ± 0.09b	0.11 ± 0.01c	0.12 ± 0.02b

Values are the mean ±  SE (n =  3). Different lowercase letters indicate significant differences according to Duncan’s test.

## Discussion

### Improvement in rice plant growth under salt stress

Salt stress is one critical factor that leads to a decline in plant growth compared with normal conditions; this is due to oxidative stress, ionic toxicity, and hormonal imbalance [37]. However, numerous studies have shown that ABA can induce plant tolerance to a variety of abiotic stresses, such as salt [38], heat [37], drought [39], low temperature [40], and heavy metals [41].

This study found that salinity stress affected the agronomic traits of both rice cultivars by decreasing their shoot and root growth morphological indexes (Figs 2–4). This was as previously found by [42]. Salt-induced reduction of plant growth is most likely due to osmotic stress arising both from low water uptake and ionic toxicity caused by excess salt entering the plant [43].

The present study also found that 1’,4’-*trans*-diol-ABA alleviated salt stress–induced reductions in the growth of the aboveground plant parts and roots, as observed in tobacco [44]. However, the trend was slightly different between the HHZ and XLY900 rice cultivars, which may have been due to their different sensitivity responses. The structure of 1’,4’-*trans*-diol-ABA is very similar to that of ABA [44], so it was speculated that it may alleviate plant growth retardation under salt stress via the same mechanism. One of the most common functions of ABA is to induce stomatal closure, thereby reducing water loss from plants and ensuring normal plant growth [45]. However, foliar spraying with 1’,4’-*trans*-diol-ABA was found to increase the *gs* in this study (Fig 6b). Thus, the mitigation effect of 1’,4’-*trans*-diol-ABA on the salt-induced decline in the growth and dry matter of the rice seedlings may not have been through the elevated triggering of the stomatal closure response, but directly improving photosynthetic efficiency to promote the accumulation of organic matter.

### Improvement in rice photosynthesis-related parameters under salt stress

Besides the changes in morphology, the second dramatic and readily measurable whole-plant response to salinity was the decrease in photosynthesis-related parameters. Salt stress decreased the photosynthetic activity (Fig 6), as observed in decreased *P*_*n*_, *gs*, *C*_*i*_, and *T*_*r*_ values. This was similar to findings for tomatoes [46], potatoes [47], and rice [48]. These decreases were likely due to a range of processes. First, reductions in photosynthesis due to stomatal factors were probably caused by osmotic effects on *gs,* and this process can happen very quickly [4]. Subsequently, stomatal closure reduced *C*_*i*_ and finally decreased *P*_*n*_ and *T*_*r*_ [49]. A decline in photosynthetic capacity weakened the carbon dioxide fixation ability of the plants, resulting in the reduction of plant biomass and the eventual decrease of dry matter [27]. At the same time, the degradation of photosynthetic pigments would further reduce photosynthetic efficiency, like HHZ (Fig 5).

However, salt stress significantly increased the *Chl a*, *Chl b*, carotenoid, or total chlorophyll content of XLY900 (Fig 5). This implied that for XLY900, the loss of photosynthetic capacity under salt stress could be unlikely attributed to chlorophyll degradation as no reduction in photosynthetic pigment content was observed, similar to the findings of [50,51]. Normally, this opposite phenomenon would be odd since photosynthesis activities and photosynthetic pigment content are closely related in most cases [38], but it is not inexplicable. The constant chlorophyll content under salt stress may be related to the constant chlorophyll content per unit leaf area. This invariability may have been due to the osmotic effect of salt on growth, producing smaller and thicker leaves (Figs 2e and h), which had the effect of concentrating chlorophyll into a smaller area compared to control leaves [49]. However, due to the small leaf size, the photosynthetic efficiency of XLY900 was still weakened. This stronger ability to adapt to salt may also be one of the reasons why XLY900 is more salt tolerant than HHZ.

The application of 1’,4’-*trans*-diol-ABA improved the photosynthesis parameters (*P*_*n*_, *T*_*r*_, *C*_*i*_, *gs*, and *Chl a*, *Chl b*, carotenoid, total chlorophyll contents) of rice ﬂag leaves in both rice cultivars under saline conditions compared to those of plants without 1’,4’-*trans*-diol-ABA application (Figs 5 and 6). The main reason behind this improvement could have been that 1’,4’-*trans*-diol-ABA, an ABA analog, accelerated the recovery of the PSII core complex from the inactivated state [52], which in turn enhanced thylakoid membrane stability and promoted photosynthetic pigment synthesis or prevented oxidative degradation to slow the decline in photosynthetic rate [53]. The changes in photosynthetic parameters were also related to the ions and hormones, as discussed in the next section.

### Alleviation of oxidative injuries under salt stress

Salt-induced oxidative stress is the toxic effect of ROS on biological structures [43]. Plants respond to salinity stress by decreasing stomatal conductance to minimize water loss. As a result, the amount of absorbed light exceeds the demand for photosynthesis [54], which affects the rate of electron transport through photosystems and results in increased ROS production [10]. This can cause lipid peroxidation in cellular membranes, DNA damage, protein denaturation, carbohydrate oxidation, and impairment of enzymatic activity [55]. The terminal products of lipid peroxidation are aggressive substances, such as MDA [33,55], which increase dramatically in rice [56], alfalfa [57], and cotton [58] under salt stress. Similar results were found in this study (Figs 7a and b). The main cell defense against oxidative injury is various antioxidant components, which contribute to the detoxication of ROS species [12]. The present study revealed that the activity of CAT, POD, APX, SOD, AsA, and GSH decreased under salt stress (Fig 7). However, leaf spraying with 1’,4’-*trans*-diol-ABA significantly increased CAT, POD, and APX activity and GSH and AsA content, and decreased MDA and soluble proteins in the leaves of both rice cultivars (Fig 8). The increased synthesis of AsA and GSH was concomitant with the up-regulation of APX-related genes, both of which contributed to the maintenance of NADP concentration for smooth photosynthetic electron transport [59,60]. This may have strengthened photosynthesis (Fig 6a). Meanwhile, decreased MDA and increased soluble protein may ultimately alleviate the salt-induced growth disorder.

### Improvement in Na^ + ^/K^ +^ of rice under salt stress

Salinity inhibits plant growth by exposing osmotic stress. To maintain normal growth, plants must re-adjust to absorb large amounts of Na^ +^ and Cl^–^ to counteract the external osmotic pressure [61]. In the present study, Na^ +^ content significantly increased after salt stress (Fig 9a). But spraying with 1’,4’-*trans*-diol-ABA, Na^ +^ levels decreased significantly in the roots of HHZ and increased in the leaves of HHZ. The decrease in Na^ +^ in the roots may have been due to an increase in the transpiration rate (Fig 6d) leading to the transport of Na^ +^ from the roots to the aboveground parts, or because the application of 1’,4’-*trans*-diol-ABA upregulated the expression of genes encoding Na^ + ^/H^ +^ antiporters to reduce the absorption of Na^ +^ by crops. Excess Na^ +^ competes for binding sites with K^ + ^, inactivating enzymes that inhibit basic cell-related metabolic functions. A low Na^ + ^/K^ +^ ratio is more conducive to enhancing plant salt tolerance, which was reflected in this study (Fig 9d). Previous studies have shown that the Na^ + ^/K^ +^ ratio is maintained by high levels of Ca^2 +^ in plants under saline conditions, which possibly functions as a signaling molecule to activate salt overly sensitive signal transduction (SOS) for regulated Na^ +^ in and out of extracellular processes, but also Na^ +^ into the vacuole [62,63]. The results of the present study were indicative of this trend (Figs 9c and d).

### Involvement of phytohormones in rice salt tolerance

In this study, NaCl resulted in the disequilibrium of phytohormones ACC, ABA, IAA, SA, N6, tZr, tZ, and cZ. As shown in Tables 1 and 2, salt stress increased ACC, ABA, IAA, SA, and tZ, while the content of other hormones decreased in both seedling varieties. Spraying with 1’,4’-*trans*-diol-ABA reversed all these changes. Plant endogenous hormones are essential growth regulators. ACC is the direct precursor of the ethylene biosynthesis pathway and salinity-induced ethylene accumulation caused by elevated production of ACC hinders rice plant growth and development [38]. Low ethylene production improves plant physiology and yield formation processes [64]. IAA and SA show mainly growth-promoting effects [43,65]. But the mechanism by which spraying with 1’,4’-*trans*-diol-ABA decreased the concentration of IAA and SA in HHZ was complicated (Table 1). In an evolutionarily conserved mechanism, phytohormone signaling mediated by ABA promotes abiotic stress tolerance; it suppresses the signaling of biotic stress-related phytohormone SA [66,67], and has the same effect on IAA [68]. Certainly, these two hormones may not have mediated the improvement in HHZ growth in this study upon spraying with 1’,4’-*trans*-diol-ABA but via the accumulation of organic matter through enhancing photosynthesis.

Since 1’,4’-*trans*-diol-ABA is very structurally similar to ABA, the relationship between the use of 1’,4’-*trans*-ABA and the change in ABA concentration, as well as the physiological and biochemical changes, is worth pondering. Previous studies confirmed that suberin is a waxy substance deposited between the cell wall and the plasma membrane under salt stress conditions, whose development is controlled by ABA and increases in response to increased endodermal ABA signaling, can regulate the influx and efflux of water and solutes, including Na^ + ^[69,70]. Consequently, after spraying 1’,4’-*trans*-diol-ABA, the increase of ABA (Table 1) in rice further led to the increase of suberin, which may be the reason for the observed decline in Na^ +^ (Fig 9a). But, unlike the common function of ABA to promote stomatal closure, after spraying with 1’,4’-*trans*-ABA, no decrease in the transpiration rate caused by stomatal closure was observed in this study (Figs 5b and d). This different effect may have been due to the increase in cytokinins (N6, tZr, tZ, cZ; Table 2) that can promote stomatal opening [71,72]. Thus, the 1’,4’-*trans*-ABA–induced increase in cytokinins reversed the effect of ABA on stomatal closure and these jointly regulated the opening of stomata with ABA. Consequently, the rice leaves maintained a large *gs*, and high *T*_*r*_ and *P*_*n*_, to ensure photosynthesis and water metabolism under salt stress, which was conducive to growth and the improvement of salt tolerance. However, this proposed mechanism applies to HHZ. Unlike HHZ, photosynthesis in XLY900 may be regulated by IAA, since IAA also regulates stomatal opening in plants [73,74].

## Conclusions

**Fig 10:**
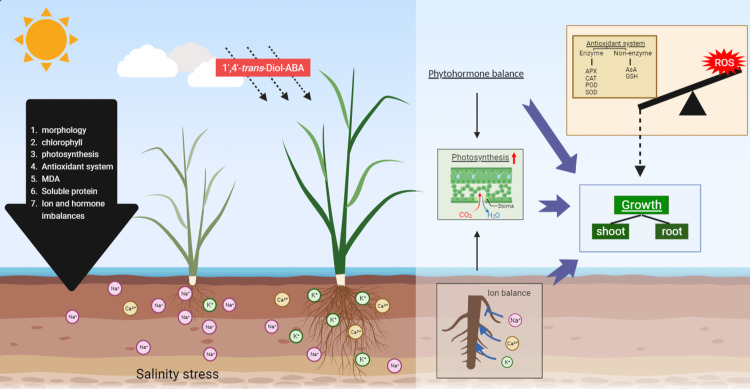
A schematic mechanism of 1’,4’-*trans*-diol-ABA in response to salt stress in rice seedlings.

This study revealed that 1’,4’-*trans*-ABA improved rice growth, as observed in the *P*_*n*_, *C*_*i*_, *gs*, *T*_*r*_, leaf area, root morphological traits, and antioxidant system, by balancing the levels of ions and hormones of the rice plants under salt stress. A schematic for the possible mechanism of 1’, 4’-*trans*-ABA in the attenuation of salt stress in rice seedlings was developed (Fig 10). 1’,4’-*trans*-ABA not only maintained the ion balance but also the homeostasis of phytohormones such as ACC, ABA, IAA, SA, and CTK. Furthermore, 1’,4’-*trans*-ABA increased the chlorophyll and carotenoid content. In the future, 1’,4’-*trans*-ABA, as an economical and environmentally friendly plant growth regulator, could be applied to improve plant growth and development. The improvements upon treatment with 1’,4’-*trans*-ABA are an essential step towards optimizing agricultural ﬁeld production on salinized land. This study showed that, in general, 1’,4’-*trans*-diol-ABA has different effects to those of ABA, indicating that 1’,4’-*trans*- diol-ABA and ABA may not be fully functionally aligned.

## Supporting information

S1 Data Raw data. (XLSX)
